# Significance of bioinformatics in research of chronic obstructive pulmonary disease

**DOI:** 10.1186/2043-9113-1-35

**Published:** 2011-12-20

**Authors:** Hong Chen, Xiangdong Wang

**Affiliations:** 1 Department of Pulmonary Medicine, Zhongshan Hospital, Fudan University, Shanghai, China; 2 Biomedical Research Center, Zhongshan Hospital, Fudan University, Shanghai, China

**Keywords:** bioinformatics, genomics, proteomics, chronic obstructive pulmonary disease, biomarkers

## Abstract

Chronic obstructive pulmonary disease (COPD) is an inflammatory disease characterized by the progressive deterioration of pulmonary function and increasing airway obstruction, with high morality all over the world. The advent of high-throughput omics techniques provided an opportunity to gain insights into disease pathogenesis and process which contribute to the heterogeneity, and find target-specific and disease-specific therapies. As an interdispline, bioinformatics supplied vital information on integrative understanding of COPD. This review focused on application of bioinformatics in COPD study, including biomarkers searching and systems biology. We also presented the requirements and challenges in implementing bioinformatics to COPD research and interpreted these results as clinical physicians.

## Introduction

Chronic obstructive pulmonary disease (COPD) is an inflammatory disease characterized by the progressive deterioration of pulmonary function and increasing airway obstruction [1,2]. It can be caused by inflammatory responses triggered by noxious particles or gas, most commonly from tobacco smoking and is accompanied by chronic bronchitis and emphysema [3,4]. Some patients go on to require long-term oxygen therapy or even lung transplantation [3]. COPD was ranked as fourth leading cause of death worldwide and is estimated to become the top third cause of mortality by 2020 [5]. According to the data in China, COPD ranks as the fourth leading cause of death in urban areas and third in rural areas[6]. The high mortality and morbidity with COPD, and its chronic progressive nature, have promoted the need to investigate the underlying mechanisms and identify biomarkers for diagnosis, prognosis and drug target.

The understanding of COPD increased by advanced molecular biology approaches, genetically modified animals, virally administered genes, and high-throughput transcriptional profiling approaches. High-throughput methodologies, such as genomics and proteomics, are commonly used. The variety of data from biology, mainly in the form of DNA, RNA and protein sequences is putting heavy demand in computer sciences and computational biology. Bioinformatics, including many sub-disciplines, such as genomics, proteomics and system biology, is an integration of mathematical, statistical, and computational methods to analyze biological, biochemical, and biophysical data. Compared to wet-lab method, bioinformatics focused on data mining via computational means. Sophisticated bioinformatics techniques are developed to analyze the vast amount of data generated from genomics and proteomics studies, such as gene and protein function, interactions and metabolic and regulatory pathways. However, there is still a great challenge to combine the computer figures with clinical data for both bench-scientists and bedside-physicians.

In COPD studies, there are usually three ways to analyze 'omics' data: 1) search correlation between single gene or protein and some clinical features in order to find diagnostic or prognostic biomarkers; 2) integrate clinical and wet-lab information, or omics data from different levels for database establishment and computational models. In this current review, we discussed application of bioinformatics in COPD study. We also presented the requirements and challenges in implementing bioinformatics to COPD research, and gave some suggestions on how to interprete these results as clinical physicians.

## Application of bioinformatics in biomarker searching

The diagnosis of COPD is based on the presence of typical symptoms of cough and shortness of breath, together with the presence of risk factors, and is confirmed by spirometry. Therefore, searching for better biomarkers with high specificity and sensitivity indicating the staging and severity of COPD remain as major concerns for clinical physicians. The main value of biomarkers in COPD would be in early diagnosis and to provide the early proof of drug efficacy during the treatment [7]. As a biomarker for COPD, it is expected to be detected in human lung fluids or tissues, sensitive to the progress of COPD, disease-specific to COPD and associated with the status of patients [8]. In these researches, selected genes or proteins usually combined with several clinical features, such as disease susceptibility, lung function, via statistical methods, i.e. logistic regression.

### Genomics

It is believed that many genetic factors increase a person's risk of developing COPD[9]. The high mortality and morbidity associated with COPD, and its chronic and progressive nature, has prompted the use of molecular genetic studies in an attempt to identify susceptibility factors for the disease. The advent of high-throughput methodologies to study genetic background variability, epigenetic regulation allowed us to explain the individual variability in the susceptibility of human diseases.

Single nucleotide polymorphism (SNP) was a common method in COPD study. These analyses usually study a group of candidate genes, and then perform statistical test in different populations. The gene-related susceptibility can be approached by testing or unbiased study designs [10]. By SNP microarray, many genetic factors were found to be related with the individual risk of developing COPD [9]. Apart from recognized deficiency of alpha1antitrypsin [9], genomics in COPD found that other gene alleles, such as IREB2[11], CYP2E1 and NAT2[12], CYP1A1, CYP1A2 and CYBA[13], TNF-α [14] were associated with COPD susceptibility. PIM3 allele of the alpha1antitrypsin gene had an association with the pathogenesis of COPD in the Indian population [15]. The polymorphisms in SP-A1 and SP-A2[16], COX2 and p53 risk-alleles [17]might be genetic factors contributing to the susceptibility to COPD. On the other hand, COPD could influence single gene expression as well, such as cathepsin inhibitory cystatin A[18].

COPD is featured by decline in lung function in disease progression. Therefore, except susceptibility, other case-control genomics studies focused on the association of several gene alleles with lung function. 105V/114V alleles of GSTP1 and 113H/139H alleles of mEPHX and the combination of genotypes with same alleles were associated with imbalanced oxidative stress and lung function in patients [19]. Polymorphisms in ADAM33 were associated with COPD and lung function decline in long-term smokers [20] and general population [21]. The variants and their combinations of eNOS -786C, -922G, and 4A alleles in endothelial cells contribute to disturbed pulmonary function and oxidative stress in COPD[22].

An interleukin13 polymorphism in the promoter region may modulate the adverse effects of cigarette smoking on pulmonary function in long-term cigarette smokers [23]. These genomics findings suggested that environmental influences were important in COPD. Genetic polymorphisms contributed to the development of COPD, especially to the declined lung function. The diversity in human genes could help us to understand the susceptibility among different ethics and different populations. The dissection of the genetic basis of complex diseases and the development of highly individualized therapies remain lofty but achievable goals [24].

### Proteomics

**Figure 1:**
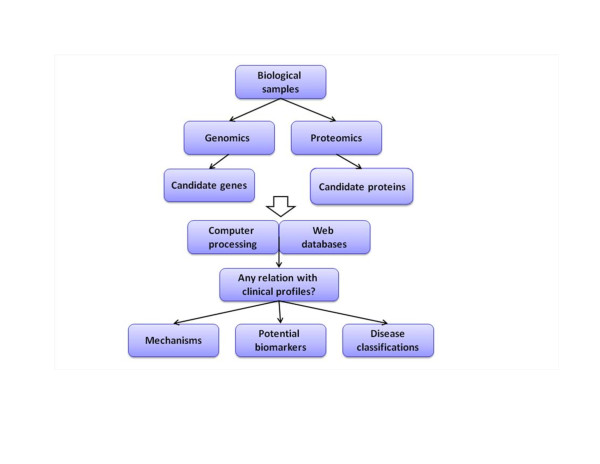
**Overview of the utility of bioinformatics in chronic obstructive pulmonary disease**. Both genomics and proteomics provide information on candidates. By searching in various datasets and combining with clinical profiles, 'omis' studies may help to explain questions on disease classification, biomarker detection, and identification of mechanism.

Proteomics is the systematic study of the many and diverse properties of protein profiles in a parallel manner with the aim of providing detailed descriptions of the structure, function and control of biological systems in health and disease [25]. A major research objective is to search for biomarkers in complex biological fluids. The proteomic analysis highlights the avenues to investigate protein profiles of cells, biopsies and fluids, explore protein-based mechanisms of human diseases, define subgroups of disease, and identify novel biomarkers for diagnosis, therapy and prognosis of multiple diseases and discover new targets for drug development. In particular, the application of complementary approaches, including gel- and liquid chromatography mass spectrometry-based proteomic techniques on sputum and/or bronchoalveolar lavage may provide a better understanding of the proteome differentially expressed among the courses, severities and populations of COPD [7]. We have previously reviewed the clinical studies on COPD proteomics, highlighted the proteomic-oriented methods applied and evaluated the diagnostic or prognostic values of potential biomarkers [8]. Those studies mainly focused on disease classification, biomarker detection, or identification of mechanism, while the three components are related with each other in COPD (Figure 1).

An important goal of proteomic studies is to understand biological roles of specific proteins and develop new therapeutic targets [26]. Although there are few proteomics studies performed in COPD patients, several potential proteins have been regarded as biomarkers. For example, matrix metalloproteinase (MMP)-13 and thioredoxin-like 2 in lungs increased in patients with COPD [27]. The serine and MMP proteinase network was considered as an important feature in predicting clinical worsening of airway obstruction [28]. Pulmonary surfactant A was found to link to the pathogenesis of COPD and could be considered as a potential COPD biomarker [29]. Proteomic screening of sputum yields potential biomarkers of inflammation [30]. Airway and parenchymal phenotypes of COPD were suggested to be associated with unique systemic serum biomarker profiles [31]. The utility of proteomic profiling would improve the understanding of molecular mechanisms involved in cigarette smoking-related COPD by identifying plasma proteins that correlate with declined lung function [32]. The concentrations of neutrophil defensins 1 and 2, calgranulin A, and calgranulin B were elevated in smokers with COPD when compared to asymptomatic smokers [33]. Other candidates, like serum amyloid A [34], plasma retinal-binding protein, apolipoprotein E, inter-alpha-trypsininhibitor heavy chain H4, and glutathione peroxidase[35], were also been detected in plasma in COPD patients by proteomics approaches.

### Metabolomics

Metabolomics is a global way to understanding regulation of metabolic pathways and metabolic networks of a biologic system[36]. Metabolites trigonelline, hippurate and formate in urinary were identified to be associated with baseline lung function of COPD patients and considered to reflect lifestyle differences affecting overall health [37]. Another way to analyze omics data is clustering data to form a specific pattern for different groups by principal component analysis (PCA). Combination of PCA and metabolomics identified the metabolic fingerprint of exhaled breath condensate of COPD patients [38].

### Multiplexed ELISA

**Figure 2:**
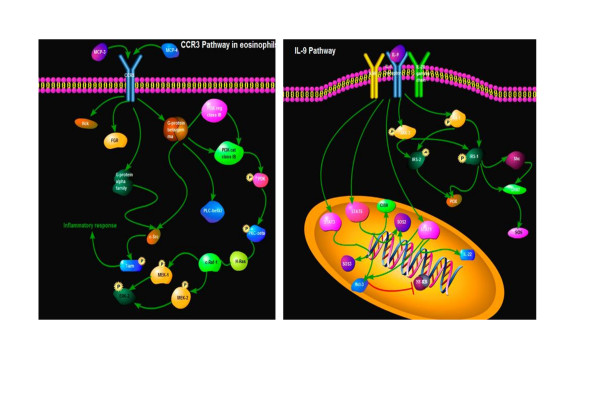
**The integration of IL-9 pathway and CCR3 pathway between biological function and pathology demonstrated cell proliferation-related remodeling, intracellular signal-associated inflammatory responses and over-activation of kinases-correlated emphysema in the pathogenesis of COPD**.

A recent advancement in ELISA is the multiplexed ELISA which could determine multiple proteins within a single tissue sample. It has recently been shown to be more sensitive than standard ELISA once optimized for a particular cytokine [39-41] and could be a promising diagnostic assay in lung diseases [42]. Moreover, the measurement of multiple cytokines is required for many diseases, particularly those like COPD that arise from a complex process of initiation and progression of inflammation network. Simultaneous detection of multiple cytokines will undoubtedly provide a more powerful tool to quantifiably measure cytokines in different stages of COPD. With help of this high-throughput measurement platform, we could integrate both biologic and clinical data to inform predictive multiscale models ranging from the molecular to the organ levels, as shown in Figure 2. For example, the integration of IL-9 pathway and CCR3 pathway between biological function and pathology demonstrated cell proliferation-related remodeling, intracellular signal-associated inflammatory responses and over-activation of kinases-correlated emphysema in the pathogenesis of COPD. We propose this new way will increase our insight into disease process and have great potential to identify new biomarkers for disease diagnosis as well as novel therapeutic targets.

## Systems biology and database establishment

The difficulties encountered whilst exploring pathogenesis and searching for biomarkers may be due in part to the complex nature of COPD, which comprises a broad spectrum of histopathological findings and respiratory symptoms [43]. All genetic information and molecular knowledge need to be semantically incorporated and associated with clinical and experimental data. System biology in COPD (as reviewed before[44]) presented a manifold understanding of the complexity of COPD, therefore advanced biomedical research and drug development. This approach relies on global genome, transcriptome, proteome, and metabolome data sets collected in cross-sectional patient cohorts with high-throughput measurement platforms and integrated with biologic and clinical data to inform predictive multidimensional models ranging from the molecular to the organ levels[45].

Comandini etc. [46] assayed a number of published studies by creating a smoker datasets on which to perform data-mining analysis. They utilized Ingenuity Pathways Analysis, a web-based application that enables identifying relationships, biological mechanisms, functions, and pathways of relevance associated with the molecules under study. Their findings supported the central role of anti-oxidant genes in smoking population and suggested Nrf2 may be a COPD risk biomarker. A brand new knowledge base was generated from clinical and experimental data for COPD based on BioXM software platform [47]. This integrated database reduced implementation time and effort for the knowledge base compared to similar systems and provided a free, comprehensive, easy to use resource for all COPD related clinical research.

Clinical profiles could also be considered as a form of omics data since it provided a large quantity of patients' information in a direct conservative way. An in-silico research applied various explorative analysis techniques (PCA, MCA, MDS) and unsupervised clustering methods (KHM) to study a large dataset, acquired from 415 COPD patients, to assess the presence of hidden structures in data corresponding to the different COPD phenotypes observed in clinical practice. This study may be considered as a methodological example showing possible applications of intelligent data analysis and visual exploratory techniques to investigate clinical aspects of chronic pathologies where a mathematical referring model is generally missing[48].

## How to understand bioinformatics as clinical physicians

COPD has been approached by genomic and proteomic technologies to allow us to identify patterns of gene/protein expression that track with clinical disease or to identify new pathways involved in disease pathogenesis. The results from these initial studies highlight the potential for these omics approaches to reveal novel insights into the pathogenesis of COPD and provide new tools to improve diagnosis, clinical classification, course prediction, and response to therapy. Existing knowledge such as genotype-phenotype relations or signal transduction pathways must be semantically integrated and dynamically organized into structured networks that are connected with clinical and experimental data. This will require collaboration among multidisciplinary groups with expertise in the respective technologies, bioinformatics, and clinical medicine for the disease. More and more clinical physicians began to realize the promise of these studies and the potential to revolutionize the diagnosis and treatment of COPD, while obstacles still existed between these laboratory findings and their applications in clinical practice. Omics results need to be interpreted by translational medicine and systems biology. Since the vocabulary of systems biology is different than that of molecular biology or clinical research, the biggest challenge is the shift in thinking[49].

Instead of a single-factor approach, which is highly effective in the lab, we need to think globally as clinical physicians. We need to shift from an approach that tries to explain lung pathogenesis by "one molecule, one cell type" to approach that looks at the network of interactions between multiple molecules, pathways, and cells. Given that all the samples were collected from human, it would be of great significance to standardize patient groups. Criteria of clinical informatics and medical informatics, including age, gender, smoking history, staging, complications and clinical signs as well as examinations, should be fully considered before and after any omics investigation. We also need to pay attention to the relation between clinical data and laboratory findings. For this to occur, a well-done history and physical examination would be helpful to supplement these laboratory figures by providing multiple features of human COPD.

**Figure 3:**
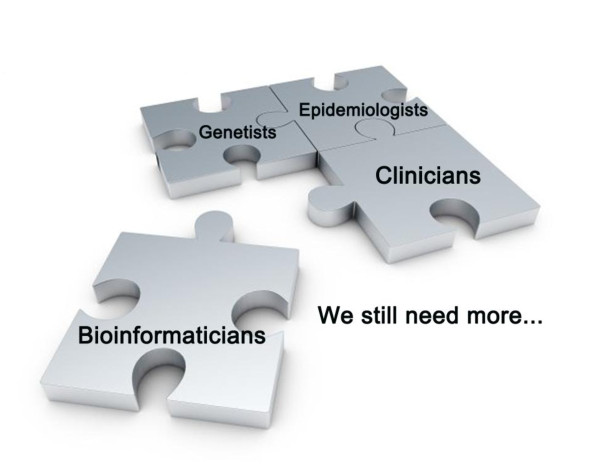
**Bioinformatics begin to take part in COPD investigation**. The collaboration of epidemiologists, clinicians, geneticists and specialists in bioinformatics, in addition to specialists in disciplines less familiar to epidemiologists, is critical for further study. More co-operations are still needed.

Although all of these exciting technological advances that exponentially increase the levels of knowledge about every disease and model serve as facilitators of integration, they do not inherently provide integrative models of disease. Therefore, we proposed that digitalizing essential clinical profiles, such as symptoms and signs, by questionnaires and/or scores, would provide direct vision for physicians and shrink the distance between lab discovery and clinical condition. The combination of epidemiologists, clinicians, geneticists and specialists in bioinformatics, in addition to specialists in disciplines less familiar to epidemiologists, is critical to be prepared for new phenotypic characterizations based on transcriptome and proteome [50]. Even though we have spotted considerable advancement in bioinformatics, it still calls for more collaboration to fulfill its potential (Figure 3). Interdisciplinary teams should allow us to access omics datasets integratively and generate a global model of COPD. It is important to have a special attention from proteomic scientists to explore the combination between advanced proteomic biotechnology, clinical proteomics, tissue imaging and profiling, and organ dysfunction score systems, to improve the clinical outcomes of these patients [51]. There is still a great need to explore the COPD-specific and/or related transcriptional factors and regulation networks generated from omics and bioinformatics like in other diseases [41].

## Conclusions

**Figure 4:**
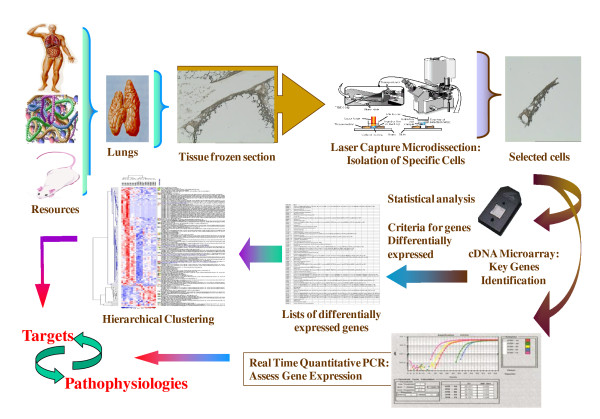
**Clinical bioinformatics can be generated from the analysis of COPD-specific pathological alterations**. Patients with COPD can be selected by clinical informatics and criteria and the specific area is selected by micro-dissection. The tissue can be used for genomic (or proteomic) analysis and identified biomarkers are validated for the understanding of the pathogenesis.

The use of high-throughput techniques for gene and protein expression profiling and of computerized databases has become a mainstay of biomedical research. There is a need to perform omics studies on patients with COPD, describing the association with the disease in terms of specificity, severity, progress and prognosis and monitoring the efficacy of therapies. These omics analysis highlight the ways to investigate protein profiles of cells, biopsies and fluids, explore protein-based mechanisms of human diseases, identify novel biomarkers for diagnosis, therapy and prognosis of multiple diseases and discover new targets for drug development, as shown in Figure 3. Although the number of clinical studies on COPD is limited, they still serve as the outstanding initiation for proteomic research in such a complex disease. The analysis of protein profiles that are up- or down-regulated, modified, secreted in the airways during the disease may yield vital evidences to understand the pathogenesis and discover new therapeutic targets for the disease (Figure 4) [52]. With many guidelines now in place and model studies on which to design future experiments, there is reason to be optimistic that candidate protein biomarkers will be discovered using proteomics and translated into clinical assays[53]. With better study design standardization and the implementation of novel technologies to reach the optimal research standard, there is enough reason be optimistic about the future of omics research and its clinical implications [54]. Clinical bioinformatics on COPD could be achieved from the combination of clinical informatics, medical informatics, bioinformatics and informatics by collaborations among clinicians, bioinformaticians, computer scientists, biologists, and mathematicians.

## Competing interests

The authors declare that they have no competing interests.

## Authors' contributions

HC mainly wrote the manuscript, as well as the revision. XDW conceived of the review and edited and wrote part of the manuscript. All authors read and approved the final manuscript.
